# Supplementary material for: Netcombin: An algorithm for constructing optimal phylogenetic network from rooted triplets
Source: PLoS One. 2020 Sep 18;15(9):e0227842. doi: 10.1371/journal.pone.0227842 (PMC7500971; doi:10.1371/journal.pone.0227842)

⊢0.0010

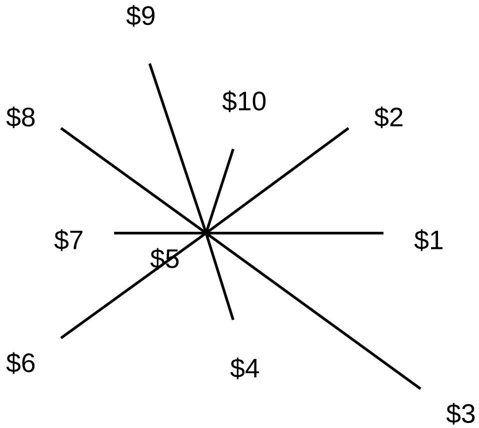

Supplement: S4 File — (ZIP) [file pone.0227842.s004.zip › SequenceData-Triplet/sequenceData-10-20-30-0-Triplet/length 200, numseq 10, rate 10^-7/1 seq.pdf]
